# An effectiveness-implementation hybrid trial of phone-based tobacco cessation interventions in the Lebanese primary healthcare system: protocol for project PHOENICS

**DOI:** 10.1186/s43058-023-00456-w

**Published:** 2023-06-26

**Authors:** Ramzi G. Salloum, Maya Romani, Dima S. Bteddini, Fadi El-Jardali, Ji-Hyun Lee, Ryan Theis, Jennifer H. LeLaurin, Randa Hamadeh, Mona Osman, Ruba Abla, Jihan Khaywa, Kenneth D. Ward, Donna Shelley, Rima Nakkash

**Affiliations:** 1 Department of Health Outcomes and Biomedical Informatics, University of Florida College of Medicine, 2004 Mowry Road, Gainesville, FL USA; 2 Department of Health Promotion and Community Health, Faculty of Health Sciences, American University of Beirut, Beirut, Lebanon; 3 Department of Family Medicine, American University of Beirut Medical Center, Beirut, Lebanon; 4 Department of Health Management and Policy, Faculty of Health Sciences, American University of Beirut, Beirut, Lebanon; 5 Knowledge to Policy (K2P) Center, Faculty of Health Sciences, American University of Beirut, Beirut, Lebanon; 6 Department of Biostatistics, College of Public Health and Health Professions and College of Medicine, University of Florida, Gainesville, FL USA; 7 Division of Quantitative Sciences, University of Florida Health Cancer Center, University of Florida, Gainesville, FL USA; 8 Ministry of Public Health, Beirut, Lebanon; 9 Division of Social and Behavioral Sciences, School of Public Health, University of Memphis, Memphis, TN USA; 10 Department of Public Health Policy and Management, School of Global Public Health, New York University, New York, NY USA; 11 Department of Global and Community Health, College of Public Health, George Mason University, Fairfax, VA USA

**Keywords:** Tobacco cessation, Primary healthcare, Low-resource settings, Implementation

## Abstract

**Background:**

Tobacco use remains the leading cause of preventable disease, disability, and death in the world. Lebanon has an exceptionally high tobacco use burden. The World Health Organization endorses smoking cessation advice integrated into primary care settings as well as easily accessible and free phone-based counseling and low-cost pharmacotherapy as standard of practice for population-level tobacco dependence treatment. Although these interventions can increase access to tobacco treatment and are highly cost-effective compared with other interventions, their evidence base comes primarily from high-income countries, and they have rarely been evaluated in low- and middle-income countries. Recommended interventions are not integrated as a routine part of primary care in Lebanon, as in other low-resource settings. Addressing this evidence-to-practice gap requires research on multi-level interventions and contextual factors for implementing integrated, scalable, and sustainable cessation treatment within low-resource settings.

**Methods:**

The objective of this study is to evaluate the comparative effectiveness of promising multi-component interventions for implementing evidence-based tobacco treatment in primary healthcare centers within the Lebanese National Primary Healthcare Network. We will adapt and tailor an existing in-person smoking cessation program to deliver phone-based counseling to smokers in Lebanon. We will then conduct a three-arm group-randomized trial of 1500 patients across 24 clinics comparing (1) ask about tobacco use; advise to quit; assist with brief counseling (AAA) as standard care; (2) ask; advise; connect to phone-based counseling (AAC); and (3) AAC + nicotine replacement therapy (NRT). We will also evaluate the implementation process to measure factors that influence implementation. Our central hypothesis is that connecting patients to phone-based counseling with NRT is the most effective alternative. This study will be guided by the Exploration, Preparation, Implementation, Sustainment (EPIS) framework, supported by Proctor’s framework for implementation outcomes.

**Discussion:**

The project addresses the evidence-to-practice gap in the provision of tobacco dependence treatment within low-resource settings by developing and testing contextually tailored multi-level interventions while optimizing implementation success and sustainability. This research is significant for its potential to guide the large-scale adoption of cost-effective strategies for implementing tobacco dependence treatment in low-resource settings, thereby reducing tobacco-related morbidity and mortality.

**Trial registration:**

ClinicalTrials.gov, NCT05628389, Registered 16 November 2022.

**Supplementary Information:**

The online version contains supplementary material available at 10.1186/s43058-023-00456-w.

Contributions to the literature

- This study will be among the first to evaluate primary care smoking cessation interventions in the Eastern Mediterranean.
- If the interventions are effective, they will inform broad dissemination to other Eastern Mediterranean countries and other low-resource settings
- This study is expected to generate unique evidence on the effectiveness and cost-effectiveness of population-based cessation interventions for waterpipe smokers.
- This study addresses an urgent need for research into implementation challenges that limit the impact of evidence-based interventions in low-resource settings to reduce the global burden of cancer.
- The expanded use of rigorous implementation science methods can be applied in other low-resource settings.

## Background

Tobacco use remains the leading cause of preventable disease, disability, and death globally due to its adverse health effects, including cancer, cardiovascular, and respiratory diseases [1, 2]. Although many regions of the world have made remarkable progress in curbing the tobacco epidemic, many low- and middle-income countries (LMICs) are lagging behind [3]. Among all World Health Organization (WHO) regions, the Eastern Mediterranean Region is making the least progress, where the number of smokers is projected to continue to increase by 2025 [3]. Lebanon, an Eastern Mediterranean country, has an exceptionally high tobacco use burden. Findings from a recent large nationally representative household survey in Lebanon indicated alarmingly high estimates of smoking prevalence: 35% of adults are current cigarette smokers, 39% are waterpipe smokers, and 4% are dual smokers [4]. Additionally, data from 2005 to 2015 collected from the National Cancer Registry of Lebanon revealed an increasing trend in lung cancer incidence, with the rate being the highest for females and the second highest for males in the Eastern Mediterranean Region [5]. Given the high burden of smoking in Lebanon, there is a persistent need to implement smoking cessation interventions.

The WHO Framework Convention on Tobacco Control (FCTC) is an international treaty that provides a framework for tobacco control measures to be implemented by its parties to continually and substantially reduce the prevalence of tobacco use and exposure to tobacco smoke [6, 7]. Article 14 of the FCTC addresses the issue of tobacco dependence treatment, defined as “the provision of behavioral support or medications, or both, to tobacco users, to help them stop their tobacco use” [8]. The following interventions have been identified as standard of practice for population-level tobacco dependence treatment: smoking cessation advice integrated into primary care settings, as well as easily-accessible and free phone-based counseling, and low-cost pharmacotherapy [8, 9]. There has been substantial progress in establishing strong scientific evidence supporting these interventions [8–10]. However, this evidence is mainly from high-income countries, and such interventions have been rarely evaluated in LMICs [11–13]. Guidelines for implementation of Article 14 recommend integrating tobacco cessation interventions into existing healthcare systems as an instrumental step towards comprehensive cessation support [8]. Although Lebanon ratified the WHO FCTC in 2005 [14], it has not prioritized implementing Article 14 and has been unable to integrate tobacco dependence treatment programs into its existing health system programs [14, 15]. This proposed project addresses the evidence-to-practice gap in the provision of tobacco dependence treatment within low-resource settings by developing and testing contextually tailored multi-level interventions while optimizing implementation success and sustainability.

As outlined in this protocol, the objective of Project PHOENICS (PHOne ENabled Implementation of Cessation Support) is to evaluate the comparative effectiveness of promising multi-component interventions for implementing evidence-based tobacco treatment in primary healthcare centers within the Lebanese Primary Healthcare Network.

More specifically, the aims of the study are to (1) adapt and tailor an existing in-person smoking cessation program to deliver phone-based counseling to smokers in Lebanon; (2) test the effectiveness and cost-effectiveness of a referral-based program that delivers smoking cessation services to primary healthcare patients; and (3) identify the multi-level determinants of implementation and sustainability using mixed methods.

## Methods/design

This protocol adheres to the Standards for Reporting Implementation Studies (StaRI) Statement (Additional file 1) [16].

### Regulatory approvals

This study was registered in ClinicalTrials.gov on November 16, 2022 (NCT05628389). The study was approved by the Institutional Review Board (IRB) at the American University of Beirut on June 3, 2022 (IRB#SBS-2022–0043) and the University of Florida on February 2, 2023 (IRB#202202537).

### Conceptual framework

**Fig. 1:**
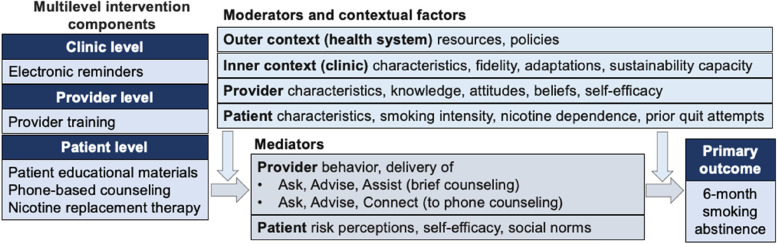
Conceptual framework

The Exploration, Preparation, Implementation, Sustainment (EPIS) framework guides this study [17]. The EPIS framework describes four phases of implementation for evidence-based interventions, as well as domains and constructs that influence the implementation process and outcomes [17, 18]. In addition to defining the phases of implementation, EPIS will guide the formative assessment (Aim 1) to ensure intervention fit and alignment with multi-level factors, including patient characteristics, provider characteristics, inner context (i.e., primary healthcare centers), and outer context (i.e., health system) (Fig. 1). EPIS will also guide the implementation process and outcomes assessment (Aim 3). To complement EPIS, the measurement of implementation outcomes in Aim 3 will be guided by Proctor’s framework for implementation outcomes [19]. Application of EPIS will optimize the relevance of findings for generalizability and scalability, and integration of Proctor’s framework will allow for the evaluation of process-based measures of implementation in relation to implementation outcomes.

### Study design overview

**Fig. 2:**
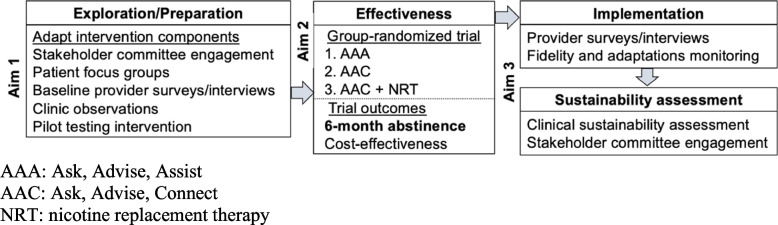
Hybrid study design overview

We propose an effectiveness-implementation hybrid (type 1) design [20] to test the comparative effectiveness of the multi-component interventions while gathering information on the multi-level factors that potentially inform the sustainment of the interventions in their setting (Fig. 2). First, we will adapt and tailor an existing in-person smoking cessation program at the American University of Beirut (AUB) Medical Center into an easily accessible phone-based counseling service for patients who smoke in Lebanon (Aim 1); we will collect pre-trial data using focus groups with patients who smoke, and we will also conduct a baseline assessment that includes surveys and in-depth interviews with providers from participating centers, and clinic workflow tailoring to further adapt the provider training and optimize the intervention fit to the primary healthcare center context. Then, we will conduct a group-randomized trial using three arms to compare the effectiveness and cost-effectiveness of the multi-component interventions (Aim 2): (1) ask about tobacco use; advise to quit; assist with brief counseling (AAA) as standard care; (2) ask; advise; connect to phone-based counseling (AAC); and (3) AAC + nicotine replacement therapy (NRT).

Table 1 presents an overview of the multilevel intervention components by arm. The proposed multi-level intervention addresses barriers to tobacco treatment at the primary healthcare center and provider levels (e.g., lack of training, lack of systems to prompt tobacco treatment) and at the patient level (e.g., nicotine dependence, social norms). To address primary healthcare center- and provider-level barriers, we will implement clinic reminders and train providers and staff on best practices, including documentation of smoking status and referral to tobacco treatment. To address individual-level barriers, all patients will receive educational materials about the harms of smoking and the benefits of cessation, patients in Arms 2 and 3 will receive phone-based counseling, and patients in Arm 3 will receive phone-based counseling and NRT. We will then conduct follow-up surveys and in-depth interviews with providers and health system administrators to identify the multi-level barriers and facilitators to implementing and sustaining tobacco treatment interventions (Aim 3). Data from Aims 1 and 3 will allow us to assess the implementation context longitudinally. This design allows us to explore the clinical infrastructure and resources needed for effective implementation.

**Table 1:** Multilevel intervention components by arm

Level	Motivating need/problem	Component	AAA	AAC	AAC + NRT
Clinic	Lack of integration into practice	Electronic reminders	◉	◉	◉
Provider	Insufficient knowledge, self-efficacy	Provider training	◉	◉	◉
Patient	Insufficient knowledge	Educational materials	◉	◉	◉
	Insufficient self-efficacy, motivation	Phone counseling		◉	◉
	Nicotine withdrawal symptoms	Nicotine patches			◉

*AAA* Ask, Advise, Assist, *AAC* Ask, Advise, Connect, *NRT* nicotine replacement therapy

### Hypotheses

Our central hypothesis is that connecting patients to phone-based counseling with NRT is the most effective alternative. We further hypothesize that (i) implementing AAA with providers will foster a supportive care climate for quit attempts, increase patient knowledge about the risks of smoking and the benefits of cessation, and modify social norms for tobacco use among smokers in Lebanon; (ii) connecting patients who smoke to phone-based behavioral counseling using AAC will increase the perceived benefits of cessation and improve norms, and promote self-efficacy among patients who smoke; and (iii) NRT will promote successful smoking abstinence by addressing nicotine withdrawal symptoms.

### Rationale for approach

Several considerations informed the selection of intervention components and targets. First, evidence on the effectiveness and cost-effectiveness of telephone counseling is not well-established in LMICs. Second, the incremental cost-effectiveness of NRT in conjunction with behavioral counseling has not been demonstrated in low-resource settings. Given the additional costs and limited access to NRT in LMICs, demonstrating the cost-effectiveness of NRT in LMICs is a critical question with implications for health system decision-makers. Third, we do not include an arm to test NRT without telephone counseling because of the evidence that behavioral support enhances the effectiveness of NRT [21, 22], and the fact that providing NRT as part of usual care in the primary healthcare center context would not be a feasible option. Finally, given the low prevalence of dual (cigarette/waterpipe) use in the recent national survey (5%) [4] and the relatively low participation of waterpipe smokers in the AUB Smoking Cessation Program (5%) [23], we anticipate that most participants will be exclusive cigarette smokers. However, we will include in our study waterpipe smokers, both exclusive and dual smokers, due to the high prevalence of waterpipe smoking in Lebanon (39%) [4], the need to identify effective interventions for waterpipe cessation, and the experience with waterpipe smokers in the AUB Smoking Cessation Program [23].

### Study setting

We will recruit 24 primary healthcare centers (each with > 500 unique patient visits annually) that are part of the National Primary Healthcare Network at The Ministry of Public Health (MoPH). The centers will be recruited from the Beirut metropolitan area, the largest in Lebanon, comprising one-third of its population, in order to optimize feasibility. Lebanon’s primary healthcare network comprises more than 250 primary healthcare centers distributed across the country and serving more than 50% of the population. The MoPH is the main authority that formulates and implements national health policies and programs. The participation of centers and providers will be coordinated through the Primary Healthcare Department at the MoPH.

### Approach for Aim 1

#### Focus groups

We will conduct eight focus groups with current tobacco users. Focus groups will follow a semi-structured guide with key questions to explore intervention adaptations informed by EPIS. Adult patients ($\ge$ 18 years) at primary healthcare centers who currently smoke cigarettes and/or waterpipe are eligible to participate in these focus groups.

#### Baseline provider surveys and in-depth interviews

We will conduct baseline surveys with all providers (physicians and nurses) in the 24 centers (*n* = 120, average 5 per center) to assess provider characteristics, provider attitudes, norms, and self-efficacy related to tobacco treatment, acceptability, and appropriateness of the proposed intervention components. In-depth interviews will be conducted with select healthcare professionals (*n* = *24*) to both explain and expand on the survey data. Survey findings will inform adaptations to the interview guide specific to the context of each center. The interviews will elicit attitudes towards and experiences in implementing tobacco treatment, potential barriers and facilitators to implementing tobacco treatment, and potential barriers and facilitators to implementing and sustaining the intervention. Interviews will be audio-recorded and transcribed verbatim.

#### Workflow tailoring

We will conduct workflow assessments via direct observation to optimize the integration of the intervention into the clinical workflow and further assess factors that may influence the adoption of the intervention. We will document current center policies, workflow, systems, and staff roles and responsibilities (in general and specifically related to tobacco treatment).

#### Pilot testing

We will pilot-test the intervention in one center. We will recruit 20 eligible patients into the most intensive intervention (AAC + NRT) to further test all components. We will follow the same procedures and methods described under Aim 2.

#### Convening and engaging the stakeholder advisory committee

We will convene an advisory committee to ensure that the research is aligned with key partners’ goals for developing an effective and sustainable model for tobacco dependence treatment in Lebanon. The committee will provide feedback at each phase of the study, offering local context for implementation and guiding dissemination activities and scaling-up of best practices in Lebanon and the region. The committee represents key partners who can serve as change agents in policymaking and clinical implementation. The committee will include the Director for Primary Healthcare and Social Health at the MoPH, two medical directors of primary healthcare centers, two nurses, and two patient advocates. The stakeholder advisory committee will be engaged in adapting and tailoring the intervention by providing feedback on the assessment tools and reviewing their findings to integrate themes and survey results into the final intervention to be tested in Aim 2.

#### Analysis

Descriptive statistics will be summarized for baseline measures overall and by arm. The pilot test with 20 patients will also be summarized in a descriptive manner. Qualitative data from the audio recordings of focus groups and interviews will be transcribed verbatim in Arabic and translated into English. The principal investigators’ and research staff’s fluency in Arabic and English will ensure conceptual equivalence [24]. The initial review of transcripts will follow a thematic content analysis approach given the structured nature of our inquiry into the specific domains surrounding the implementation context [25], using a framework method for rapid and multi-disciplinary assessment of key findings [26]. We will independently use a transcript summary template to abstract findings and generate a descriptive focus group-by-theme matrix for focus groups and center-by-theme matrix for interviews, developed with primary thematic categories based on the interview questions [27]. Review of completed matrices in team meetings will resolve discrepancies, establish inter-rater reliability, and inform the development of codebooks for in-depth analysis. The in-depth analysis of qualitative data will be conducted by trained coders using iterative stages of deductive and inductive coding, enabling identification and description of emerging themes [28]. Qualitative analyses of focus groups will include deductive codes to identify potential adaptations to intervention components and functions (e.g., counseling and referral practices). Qualitative analyses of interviews will involve within-center and cross-center analysis. Selective member checking will be conducted to enhance validity.

### Approach for Aim 2

#### Patient eligibility

Eligible patients will be adult ($\ge$ 18 years) patients from the participating centers who have visited the center in the past 6 months, are daily smokers ($\ge$ 5 cigarettes or $\ge$ 1 waterpipe session per day), are reachable by phone, are interested in quitting, live in Greater Beirut and are able to provide informed consent. We will include exclusive cigarette, exclusive waterpipe and dual cigarette/waterpipe smokers. We will exclude patients who are pregnant or nursing, patients for whom NRT is medically contraindicated and those enrolled in other tobacco treatment programs.

#### Recruitment and randomization

Permuted block randomization with will be used to randomize the centers. We will randomize at the center- rather than individual-level to test the effects of multi-level intervention components while preventing the threat of contamination within a center. We plan to enroll 1500 patients and estimate that we will be able to meet recruitment goals (average 75 patients per center in the AAC and AAC + NRT arms) within 9 months in each of the eligible centers. All participants will be offered remuneration at the 6-month assessment.

#### Sample size and power evaluation

Justification of the sample size is based on two-level hierarchical mixed-effects logistic regression. The analysis aims to compare the abstinence rate at 6 months post-intervention among the three intervention arms. In this design, the subjects are the level 1 units, and the centers are the level 2 units. All subjects in a particular center receive one of the possible interventions selected at random. Power is evaluated based on the differential effect size between the AAC + NRT and AAC arms, which is expected to be the smallest difference in the abstinence rate. Sample sizes of 480 in the AAC + NRT arm and 480 in the AAC arm, which are obtained by sampling 8 centers per arm with an average of 60 subjects per center, achieve 84% power to detect a difference in the abstinence proportions of 12%. The abstinence proportion at 6 months among AAC + NRT participants is assumed to be 32% under the alternative hypothesis, while the proportion among AAC participants is 20%. The test statistic used is the effect regression coefficient from a mixed-effects logistic regression model. The intra-cluster correlation coefficient is assumed to be 0.01 [29, 30], and the significance level of the test is 0.017 after Bonferroni adjustment. Considering the expected low abstinence rate in the AAA arm and a significant difference compared to both the AAC alone and AAC + NRT arms, and weighing ethical considerations to offer the more intensive intervention when possible, 240 subjects (an average of 30 subjects) per center from 8 centers will be necessary for the AAA arm to achieve more than 85% power with a 0.017 significance level. A total of 1,200 subjects will be necessary: 480 subjects (AAC + NRT; 60 subjects from 8 centers); 480 subjects (AAC; 60 subjects from 8 centers), and 240 subjects (AAA; 30 subjects from 8 centers). Considering a 20% drop-out rate, 1500 subjects will be enrolled.

#### Measures

Measures for aim 2 are summarized in Table 2. Research assistants will administer the screener and baseline survey at the time of enrollment in the center. Follow-up assessments at 1 and 3 months will be completed by the counselor via telephone. The 6-month assessment will be completed in-person, at the center, by the research assistant to validate self-reported abstinence. All patient assessments will be administered using REDCap.

1. The primary outcome is the 7-day point prevalence (defined as any smoking, even a puff) carbon monoxide-confirmed (< 8 ppm) smoking abstinence assessed at 6-month follow-up, in line with recommendations from the Society for Research on Nicotine and Tobacco for assessing outcomes in smoking cessation trials [31].
2. Other cessation outcomes: we will include self-reported measures for continuous abstinence between baseline, 3 months, and 6 months; 24-h quit attempts; and reduction in smoking at 1, 3, and 6 months [31].
3. Multi-level mediators and moderators: patient assessments will also collect data on potential mediators (risk perceptions [32], self-efficacy [33], and social norms [34]), and moderators (age, sex, education, cigarettes/waterpipe per day, nicotine dependence [35], and prior quit attempts). Provider assessments will also collect data on center characteristics (e.g., priority, climate) and provider knowledge, attitudes, and self-efficacy [36, 37].
4. Cost: we will collect data on estimates of the time spent delivering the interventions using the provider surveys and counselor electronic logs. We will develop templates to capture these data prospectively and improve cost assessment accuracy. We will assess costs to deliver the interventions, including personnel resources based on MoPH salaries, NRT, educational materials, and technology costs. Costs of developing training materials will also be included. Any hardware and material costs necessary for implementing the interventions will be tracked through invoices. Intervention costs will exclude research costs.

**Table 2:** Aim 2 evaluation

	Measures description	Instrument/data source	Timing
Primary outcome	Carbon monoxide-confirmed abstinence	Follow-up patient visit	6 months
Other cessation outcomes	Continuous abstinence, quit attempts, smoking reduction	Phone assessment	1, 3, 6 months
Patient-level mediators	Risk perceptions, self-efficacy, social norms	Phone assessment	0, 1, 3, 6 months
Provider-level mediator	Provider behavior, delivery of AAA, AAC	Patient survey	Index visit
Patient-level moderators	Age, sex, education, cigarettes/waterpipes per day, nicotine dependence, prior quit attempts	Patient survey	Index visit
Clinic- and provider-level moderators	Clinic characteristics (priority, climate), provider knowledge, attitudes, self-efficacy	Provider interview/survey	Pre-trial
Cost	Intervention delivery costs	Cost survey/electronic log	Trial

*AAA* Ask, Advise, Assist, *AAC* Ask, Advise, Connect

#### Analysis

All analyses in aim 2 will account for the multi-level nature of the group-randomized trial (i.e., patients nested within centers). Generalized linear mixed-effects models (GLMM) will form the basis of comparisons across intervention arms, including a random effect for the center. Models of binary outcomes (primary outcome and other cessation outcomes) will use a logit link to estimate relative risks to compare arms; models of continuous outcomes (e.g., quit attempts) will use an identity link assuming a Gaussian distribution, which will be verified to compare means. While balance across arms is expected, we will examine other covariates in the models as necessary, including sex as a biological variable. We will conduct pairwise comparisons to evaluate significant differences between arms, adjusting for multiple comparisons via the step-down Bonferroni method. The multi-level GLMM approach will also be used to evaluate the interventions’ causal direct and indirect effect estimates with mediator variables. Causal mediation analysis will be conducted to decompose the effects of mediators: total effect on the odds ratio scale = direct effect × indirect effect; total effect on the excess relative risk (ERR) scale = direct effect + indirect effect; and proportion mediated on the ERR scale = indirect effect/total effect × 100% using the counterfactual framework [38–42]. SAS CAUSALMED procedure will be used to estimate causal mediation effects. All causal models will include moderator variables. A sensitivity analysis will evaluate if the intervention effects differ by smoking type (cigarettes and waterpipe). The intra-cluster correlation coefficient will be estimated from the final model to inform future research.

Stochastic imputation for handling missing data will be employed as needed [43]. Data analysis with missing data will focus on minimizing bias, maximizing use of available data, and obtaining appropriate estimates of uncertainty. Specifically, we will use intent-to-treat analysis and multiple imputation for the primary outcome, secondary outcomes, and key covariates. Multiple imputations will be applied via PROC MI/MIANALYSE in SAS and several R packages under the missing-at-random (MAR) assumption. We will consider missing data patterns (monotone, univariate, file matching) and variable type (numeric, character) in determining the imputation method. The imputation model will be consistent with our analytic model of the outcomes. If necessary, we will identify potential auxiliary variables to increase the power of our imputation model [44]. Sensitivity analyses will be performed to assess alternative multiple imputation techniques. Finally, several diagnostic statistics and graphical illustrations will be evaluated to assess imputation performance, including a fraction of missing information, relative efficiency, and trace plots.

The cost-effectiveness analysis (CEA) will estimate the incremental cost of delivering the more intensive interventions using cost-per-quit at 6 months as the main outcome. CEA will follow the recommendations of the 2nd U.S. Panel on Cost-Effectiveness in Health and Medicine [45]. Conventional CEA summarizes findings as an incremental cost-effectiveness ratio (ICER) [46]. The ICER estimates the additional resource consumption needed to achieve an increase in an additional unit of effectiveness. The ICER is then compared with a threshold value [47] to determine if the intervention is cost-effective. The net benefit approach calculates the net benefit of an intervention [48, 49]. The strategy that yields the highest net benefit is considered the most cost-effective. Another way to report the results of CEA is cost-effectiveness acceptability curves [50, 51] which estimate the probability that a new intervention yields the largest net benefit. Conventional ICER, net benefit, and acceptability curves will all be reported to facilitate comparison with other CEA in the literature. Although the societal perspective is recommended for CEA [46], our goal is to provide economic data to key stakeholder groups. Therefore, CEA will be conducted separately from the societal and health system perspectives, with appropriate adjustment to the cost measures for each perspective.

### Approach for Aim 3

#### Measures

Measures for aim 3 are summarized in Table 3. The provider surveys and in-depth interviews described in Aim 1 will be repeated post-trial to collect follow-up measures to the baseline assessments, capture adaptations to the interventions, and evaluate changes to the implementation context. Attrition will be addressed through surveys administered to additional providers at centers with missing follow-up data.

1. Treatment fidelity: to examine the fidelity of the provider-level intervention component, we will assess provider training attendance and quality using training logs and evaluations. At the patient level, we will assess the receipt of AAA or AAC as reported by the patient, the number of phone counseling sessions completed and content covered, and NRT use in the AAC + NRT arm. Patient baseline and follow-up surveys will assess the receipt of AAA or AAC components, as applicable. The telephone counselors will assess the number of sessions completed and the duration of each session. They will assess NRT use in the AAC + NRT arm in follow-up phone calls and reasons for non-adherence using the Medication Adherence Questionnaire [52], and any potential NRT use not provided by the trial in the AAA and AAC arms. To assess the intervention dose received by patients, each component will be scored 0 if the full dose is not received and 1 if received (e.g., did not complete/completed each counseling session). A fidelity score will be calculated by summing the individual dose scores (range 0–3). In addition, research assistants will observe a random sample of the phone counseling sessions and document the delivery of essential components of the counseling intervention using the Motivational Interviewing Treatment Integrity coding manual [53]. Observations will be reviewed with the phone counselors to ensure high fidelity.
2. Adaptations: follow-up interviews with the medical director and nurses most involved with the implementation process will include an assessment of adaptations using the Framework for Reporting Adaptations and Modifications-Enhanced (FRAME), a checklist and coding system for reporting adaptations and modifications made to evidence-based interventions [54, 55]. Interviews will assess the following domains related to adaptation, with follow-up open-ended questions in the interviews, as needed: (1) when and how in the implementation process the modification was made; (2) whether the modification was planned/proactive or unplanned/reactive; (3) who determined that the modification should be made; (4) what was modified; (5) at what level of delivery the modification was made (i.e., patient, provider, center); (6) the type or nature of context-level modifications; (7) the extent to which the modification was fidelity-consistent; and (8) reasons for the modification, including (a) the intent or goal of the modification and (b) the contextual factors that influenced the decision. A binary measure will be created for each center to indicate whether adaptations were reported.
3. Sustainability capacity: follow-up provider surveys will assess sustainability capacity using the clinical sustainability assessment tool (CSAT) [56], which measures the organizational factors hypothesized to impact the implementation and sustainability of evidence-based interventions. The CSAT was designed for clinical settings with 35 statements nested into 7 domains: engaged staff/leadership, engaged stakeholders, monitoring/evaluation, planning/implementation, outcomes/effectiveness, workflow integration, and organizational context/ capacity [56].
4. Implementation context and outcomes: the follow-up interviews with the medical director or chief nurse *(n* = 24) will involve the assessment of multi-level barriers and facilitators to implementation and sustainability, organized according to the EPIS framework into the outer setting (e.g., system resources, policies), inner setting (clinic priorities, care climate), and provider (e.g., attitudes towards the intervention), as well as Proctor framework implementation outcomes of acceptability, appropriateness, and sustainability. Similar in-depth interviews will be conducted with MoPH administrators (*n* = 5)—e.g., Director General, Director of the National Tobacco Control Program, Director of Information Technology.

**Table 3:** Aim 3 evaluation

	Measures	Instrument/data source	Timing
Fidelity	Provider training attendance and quality	Training log/evaluations	Pre-trial
	Patients received Ask, Advise, Assist or Ask, Advise, Connect	Patient surveys	0, 1 month
	Counseling sessions completed, content	Phone log/checklist	Weeks 1–8
	Patient used 4-week NRT. Fidelity score, sum 2–4 (range 0–3)	Patient log	1 month
Adaptations	Timing, planning level, decision maker, modification, delivery level, context, content, fidelity, reason. Adaptation occurred (0/1)	Provider interviews and surveys	Pre-/post-trial
Sustainability capacity	Staff and leadership, stakeholders, monitoring/evaluation, planning/implementation, outcomes/effectiveness, workflow integration, organizational context/capacity. Overall score (range 1.0–7.0)	Provider surveys	Pre-/post-trial
Implementation context and outcomes	Outer setting (system resources, policies), inner setting (clinic priority, climate), provider (attitudes, knowledge, self-efficacy), implementation outcomes (acceptability, appropriateness)	Health admin. interviews	Post-trial
		Provider interviews and surveys	Pre-/post-trial

*NRT* Nicotine replacement therapy

#### Analysis

We will use an explanatory sequential mixed methods approach [28] to identify the multi-level determinants of implementation and sustainability according to EPIS [17] and Proctor’s framework for implementation outcomes [19]. Our approach will follow an expansion function (with the qualitative interviews helping to expand on quantitative survey findings) and a complementarity function (with the interviews providing context around the variability observed in surveys) [57]. Given the small sample of centers and providers, we will use descriptive statistics to summarize the quantitative data from the pre- and post-trial surveys to assess provider- and center-level measures. To examine the effects of fidelity, each of the individual measures will be analyzed using the univariate GLMM approach as described for aim 2, using logit link function for the binary outcome to evaluate the effect of the interventions. Then, multivariate GLMM will be fitted for the four measures while accounting for the correlations among the measurements within the same subject. Adaptation and sustainability capacity scores will be summarized with descriptive statistics and box plots by center and arm.

Qualitative data collected from in-depth interviews with primary healthcare providers and MoPH administrators will provide additional context to patient and provider survey responses. Given that we will have collected quantitative data before qualitative data, if there are findings that necessitate explanation, we will use the qualitative interviews to explain and expand on quantitative results. Similar to aim 1, qualitative data from the audio recordings of interviews will be transcribed verbatim in Arabic and translated into English. Similarly, interview transcripts will be reviewed using the content, framework, and in-depth analysis approaches. For primary healthcare provider interviews, additional stage-by-theme matrices will be developed (for each center individually, and in the aggregate) to understand changes to implementation context between baseline and follow-up. The final product will be a theoretically informed, empirically grounded model of the multi-level factors associated with implementation of the evidence-based tobacco treatment interventions.

## Discussion

This study addresses a critical need to evaluate the comparative effectiveness and cost-effectiveness of multi-level strategies that integrate smoking cessation into clinical practice in low-resource settings. It builds upon an existing evidence-informed in-person smoking cessation program at a medical center in Beirut, Lebanon, and tailors it to a phone-based counseling intervention to smokers in Lebanon.

Considering the strong evidence that combining behavioral counseling and pharmacotherapy is more effective than either alone [22], that directly connecting smokers to phone-based counseling is more effective than standard referral [58], and that access to NRT in Lebanon and other low-resource settings is often limited [59], we will conduct a group-randomized trial of 24 centers to compare three arms, AAA, AAC, and AAC + NRT.

We expect to encounter a few potential challenges, which we have planned for alternative approaches. We might face difficulties in recruiting centers and patients. The team has tried to minimize this risk by securing buy-in for this project at multiple levels, including from MoPH leadership and medical directors at the centers. The study team is also leveraging a history of successful collaboration with the MoPH and the primary healthcare center network [60–63]. Though we expect to meet our recruitment goal, we will recruit additional centers if we are unable to achieve our desired sample from the initial 24 centers. Low fidelity is another potential challenge. In such case, we will provide booster training sessions for providers and center staff and/or telephone counselors, as needed.

This study is significant for its potential to guide large-scale adoption of tobacco treatment guidelines in the Lebanese primary healthcare network as a model for similar efforts to improve preventive practices in clinical settings across other Eastern Mediterranean countries, other LMICs, and other low-resource settings. Specifically, it addresses an urgent need for research into implementation challenges that limit the impact of evidence-based interventions in low-resource settings to reduce the global burden of cancer. The expanded use of rigorous implementation science methods can also be applied in other LMICs and in low-resource settings.

The study will have a significant public health impact as it is expected to provide a replicable model for population-based tobacco treatment interventions in low-resource settings. Prior research suggests that improving the effectiveness of such interventions requires a multi-level approach that addresses both the context of care and the barriers to behavior change [21, 58, 64, 65].

## Supplementary Information

**Additional file 1.** Standards for Reporting Implementation Studies: the StaRI checklist for completion.

## Data Availability

No study data have been collected yet. Upon study completion, any datasets used and/or analyzed during the current study will be available from the corresponding author (RGS) on reasonable request.

## Abbreviations

AAA: Ask about tobacco use; Advise to quit; Assist with brief counseling
AAC: Ask about tobacco use; Advise to quit; Connect to phone-based counseling
AUB: American University of Beirut
CEA: Cost-effectiveness analysis
CSAT: Clinical sustainability assessment tool
EMR: Eastern Mediterranean Region
EPIS: Exploration, Preparation, Implementation, Sustainment framework
ERR: Excess relative risk scale
FCTC: Framework Convention on Tobacco Control
FRAME: Framework for Reporting Adaptations and Modifications-Enhanced
GLMM: Generalized linear mixed-effects models
ICER: Incremental cost-effectiveness ratio
IRB: Institutional Review Board
LMICs: Low- and middle-income countries
MAR: Missing-at-random
MoPH: Ministry of Public Health
NRT: Nicotine replacement therapy
StaRI: Standards for Reporting Implementation Studies
WHO: World Health Organization
